# The Dancers’ Visuospatial Body Map Explains Their Enhanced Divergence in the Production of Motor Forms: Evidence in the Early Development

**DOI:** 10.3389/fpsyg.2019.00768

**Published:** 2019-04-10

**Authors:** Massimiliano Palmiero, Luna Giulianella, Paola Guariglia, Maddalena Boccia, Simonetta D’Amico, Laura Piccardi

**Affiliations:** ^1^ Cognitive and Motor Rehabilitation and Neuroimaging Unit, IRCCS Fondazione Santa Lucia, Rome, Italy; ^2^ Department of Biotechnological and Applied Clinical Sciences, University of L’Aquila, L’Aquila, Italy; ^3^ Life, Health and Environmental Science Department, University of L’Aquila, L’Aquila, Italy; ^4^ Department of Human Science and Society, Kore University of Enna, Enna, Italy

**Keywords:** dance, divergent thinking, creativity, visual, motor, verbal, education, expertise

## Abstract

Dance represents an opportunity to promote not only motor skills but also other cognitive functions and general well-being. In this study, 58 children aged 6–10 years were enrolled in order to test the issue if dance improves divergent thinking in motor and visual domains (domain-general and domain-specificity hypotheses), and whether the topological map of the body mediates their performance at the motor task (mediation hypothesis). Therefore, 33 children practicing dance were compared with a control group (25 children). Children were administered the visual divergent thinking task of the Torrance Test of Creative Thinking, consisting in making drawings starting from given shapes, and the motor-form divergent thinking task, opportunely developed consisting in producing acted motor forms in the extrapersonal space. Both tasks were presented for 10 min and were scored in terms of fluency, flexibility, and originality. The ability to form the topological map of the body was measured by the frontal body-evocation test. Results revealed that children practicing dance outperformed the control group only in terms of the ability to perform motor forms. In addition, dancers showed a better topological map of the body, and, most importantly, besides the direct effect of group on the ability to produce acted motor forms, a significant indirect effect of the group, mediated by performances on frontal body-evocation task, was found. These results have important implications for cognition, showing that dance can improve the topological map of the body that in turn enhances creativity in motor domain since the early developmental age.

## Introduction

Positive effects of physical activities on different cognitive aspects have been widely documented over the years. For example, agonistic athletes are faster at mentally rotating objects (Ozel et al., 2002), whereas individuals who performed orienteering and geocaching improved their visuospatial skills (Barnikel et al., 2014; Ellbrunner et al., 2014; Schmidt et al., 2016). Physical activity produces benefits also on child development (e.g., Iivonen et al., 2013; Zeng et al., 2017), increasing the possibilities of learning at school (Singh et al., 2012; Hillman and Biggan, 2017). In particular, dance promotes motor development and communicative skills in children (Pavlidou et al., 2018).

A growing number of studies has also demonstrated that dance improves kinetic consciousness and by consequence motor creativity and self-expression (Kalliopuska, 1989; Castañer et al., 2016; see for a review, Pavlik and Nordin-Bates, 2016), regardless of age. Indeed, the use of motor imagery to create new movements enhances creativity in dancers of all ages (Purcell, 1990; Sacha and Russ, 2006; Couillandre et al., 2008). However, the extent to which dance can improve creativity and divergent thinking in other domains has to be still clarified. Specifically, divergent thinking measures the creative potential, reflecting the ability to find many different solutions to the same open-ended problem (Guilford, 1950, 1967; Guilford et al., 1978). For instance, the Alternate Uses Test (Guilford et al., 1978) requires one to think of different uses for a common object (e.g., a brick can be used as a weapon) and is scored in terms of fluency (responses provided in a given time), flexibility (categorical shifts in responses), originality (the extent to which responses are infrequent), and elaboration (details provided along with the responses). Basing on Guilford’ seminal work, different divergent thinking tests were developed, in visual and verbal forms (e.g., Torrance, 1987), as well as in motor forms (e.g., Wyrick, 1968; Castañer et al., 2009; Scibinetti et al., 2011; Moraru et al., 2016), which are based on the production of as many actions as possible (e.g., stepping, skipping).

Well, on the one hand, modern/contemporary dancers, who basically freely improvise on stage, were found to exhibit high levels of divergent thinking in verbal and figural domains, followed by jazz/musical and then by ballet dancers (Fink and Woschnjak, 2011). In addition, professional ballet/modern dancers were found to produce higher ideational fluency than novices in verbal divergent thinking as measured by the alternative uses task (Fink et al., 2009). These results suggest that differences in divergent thinking depend on the type of dance practiced. Dance improvisation seems to enhance the creative potential more than classical dance. Even in non-professional dancers, short periods of dance improvisation (5 min) increased emotional well-being, which in turn produced higher fluency of verbal divergent thinking (Campion and Letiva, 2014). On the other hand, investigating the relation between creative ability in choreographic dancers and selected attributes of verbal and visual divergent thinking, Brennan (1982) found that measures of verbal and visual divergent thinking were not related to the measures of creative dance, suggesting that originality and flexibility of movements represent distinct divergent production abilities. Stinson (1993) also showed that students in Chinese dance education produced significantly lower scores in fluency, flexibility, and originality of visual and verbal divergent thinking than a non-dancing control group.

Although these results are contradictory, dance—as a form of art—undoubtedly involves creativity and divergent thinking, specifically in the motor domain (Alter, 1999). In this vein, several studies suggested that creativity (e.g., Plucker and Beghetto, 2004; Kaufman and Baer, 2005; Silva et al., 2009; Palmiero et al., 2015; Palmiero et al., 2016b) and divergent thinking (Palmiero et al., 2010; Boccia et al., 2015) are domain-specific. Palmiero et al. (2010) found that divergent thinking in verbal domain is mostly domain-specific, but can also be affected by general processes that underpin visual skills, such as vividness of visual mental imagery, whereas divergent thinking in visual domain is exclusively domain- and task-specific. However, elsewhere, the view that divergent thinking presumably fosters creativity across domains is supported (Silvia, 2008a,b).

On the other hand, several studies demonstrate that practicing a sport produces changes not only in cognitive and motor skills, but also on body awareness. For example, Fonseca et al. (2014) demonstrated that when 15 ballroom dancing beginners received a 1-week training per 3 months, they subsequently produced an increase of body awareness with respect to 15 controls matched for age who received no training in dance but only theoretical lessons about body perception. These results are in line with Damasio (2010), who suggests that movements allow to build up new cortical maps, increasing the repertoire of new possible motor responses. More specifically, the topological map of the body is a dynamic mental representation relevant for action and integration with the environment (Paillard, 1980) coming from tactile, proprioceptive, and kinesthetic information, as well as from environmental stimuli (Schwoebel and Coslett, 2005; Cardinali et al., 2009; Vignemont, 2010). Thus, dancing improves body awareness (e.g., Fonseca et al., 2012, 2014) on the basis of the movements of the body parts, activating the proprioceptive receptors and postural sensitivity, and informing the brain about the position of the trunk and segments (Thurm et al., 2011). Creative dance movement seems to increase also satisfaction with body appearance, fitness, and body parts, especially in experienced groups of dancers (Lewis and Scannell, 1995).

With this in mind, in the present study, we aimed at clarifying the extent to which dance can improve motor and visual divergent thinking in child development, and if the topological map of the body mediates motor divergent thinking in dancer children. We enrolled children 6–10 years old because as stated in developmental literature, childhood involves significant physical and cognitive changes including: body changes (i.e., weight; height; muscular tone and mass), motor activity (motor coordination and goal finalized actions) and logical processing of reasoning (Piaget, 1952). According to Piagetian developmental theory, children start to reason in terms of theories and abstractions, as well as concrete realities. They are capable of creating logical structures that explain their physical experiences. Not least at this developmental stage corresponds the beginning of the formal education, and different studies demonstrated that at this age, motor activity, including dance, has positive effects on learning (Singh et al., 2012; Hillman and Biggan, 2017).

According to Whitehead (2010), “physical literacy” is an opportunity to generate significant benefits for both specific- and cross-domain learning. Here, we compared children with and without dance experience in visual and motor divergent thinking. Visual divergent thinking was measured by the completion of drawing task included in the battery of Torrance Test of Creative Thinking (Torrance, 1987). With respect to motor divergence, a motor task aimed at measuring the ability to produce different motor forms (e.g., the body posture that a football player takes while kicking the ball), rather than different actions per se (e.g., the action to kick the ball), was opportunely developed. We also assessed whether motor divergent thinking skills were mediated by body representation, which was tested by asking children to form the topological map of the body task (TMB; Daurat-Hmeljiak et al., 1978; Cannoni and Tega, 2009; Di Vita et al., 2019). The novelty of this study is two-fold: first, it explores the extent to which dance promotes motor and visual divergent thinking in children; second, it assesses if the topological map of the body mediates motor divergent thinking in dancer children.

Basing on previous findings, hypotheses were formulated as follows:

1. the dance background improves both visual (e.g., Fink et al., 2009; Fink and Woschnjak, 2011) and motor divergent thinking (domain-general hypothesis);
2. the dance background has a specific effect only on motor divergent thinking (e.g., Brennan, 1982; Stinson, 1993) (domain-specific hypothesis);
3. the ability to form the topological map of the body is better in dancers than non-dancers (e.g., Fonseca et al., 2012, 2014) on the basis of a better postural sensitivity and body awareness;
4. the improvement in motor divergent thinking is mediated by the improvement in topological map of the body in dancers (mediation hypothesis).

## Materials and Methods

### Participants

A total of 58 children participated in this study (age range: 6–10 years). They were subdivided into two groups as follows: the experimental group was composed by 33 ballet dancers (22 girls and 11 boys; mean age = 8.4 S.D. ± 1.27; dance expertise in years = 2.94 S.D. ± 1.50) of the different gyms belonging to the amateur sportive association “The Starlight Company” located in Rome; the control group was composed by 25 non-dancers (11 girls and 14 boys; mean age = 7.92 S.D. ± 1.50) of the same gyms. The two groups did not differ in age (*F*_(1,56)_ = 1.69, *p* = 0.19). The non-dancers practiced other types of physical activities (e.g., volleyball and football). All children were Italian native speakers and attended the primary school. Children with learning difficulties and other neurodevelopmental diseases were not included in the sample. As reported by parents during an informal interview, children had no primary visual or hearing impairments, no neurological conditions, and no emotional or behavioral problems. The study was approved by the Ethics Committee of University of L’Aquila in accordance with the Declaration of Helsinki. A signed informed consent was obtained from parents and an assent from each child.

## Materials and Procedure

### The Topological Map of the Body Task (Daurat-Hmeljiak et al., 1978; Lis and Tallandini, 1981, for the First Italian Standardization and Cannoni and Tega, 2009, for an Updating of the Italian Normative Data)

**Figure 1:**
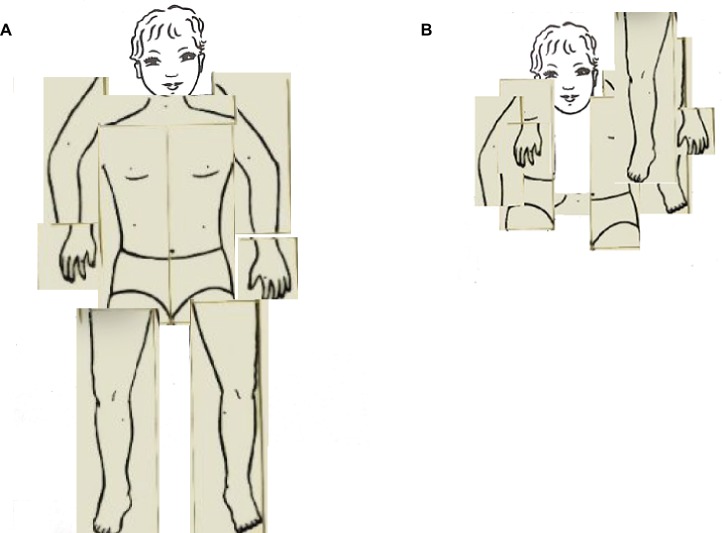
The topological map of the body: **(A)** Good; **(B)** Wrong.

The topological map of the body (TMB) was assessed using the Frontal body-evocation subtest (FBE) of the Body Representation Test (Daurat-Hmeljiak et al., 1978; Cannoni and Tega, 2009). This task is still largely used in its original and adapted versions both in clinical and experimental settings over the life span to study alterations in the mental representation of the relations between body parts (e.g., Guariglia et al., 2002; Marangolo et al., 2003; Di Russo et al., 2006; Canzano et al., 2011; Cimmino et al., 2013; Fuentes et al., 2013; Palermo et al., 2014; Bassolino et al., 2015; Di Vita et al., 2015, 2017; Spitoni et al., 2015; Zantedeschi and Pazzaglia, 2016; Perez-Marcos et al., 2018). Task materials included a small plastic board on which the position of the head was depicted as a reference part, and nine tiles, each representing a body part. Children were presented with one tile at a time and their task was to name the body part depicted on the tile; then, they were instructed to place each tile on a board, on which was depicted only a child’s face. Before showing a new tile, the position of the previous tile was recorded by overlapping a transparent grid and the tile was removed. The number of correct answers was recorded (Max = 9) as well as the response time expressed in seconds (see Figure 1).

### The Visual Divergent Thinking Task (From Torrance Test of Creative Thinking—Form A (Torrance, 1987); Italian version, Sprini and Tomasello, 1989)

This task asked children to make drawings starting from given shapes within 10 min. Basically, a paper sheet with 10 incomplete shapes was presented; children were told to complete the shapes adding details and providing titles along with each drawing. Children could also use colored pencils to make their drawings. This task is also largely used in literature (e.g., Ayob et al., 2012; Palmiero et al., 2016a, 2017; Humble et al., 2018). It allows to assess three basic attributes of visual divergent thinking. First of all, fluency, that is the number of appropriate ideas/drawings provided within a time limit. Secondly, flexibility, that is the number of categories encompassing the ideas underlying the drawings. As reported in the technical manual, the list of categories for each starting shape covered about 99% of the responses provided by the reference sample, formed by 500 people; when the category was not present, the scoring procedure allowed to opportunely generate a new category. The sum of the categories across responses was used as the individual flexibility score. Finally, originality, that is the number of statistically infrequent ideas provided by a reference sample reported in the technical manual: responses provided by 5% or more of 500 people were scored 0 for originality; responses provided by 2–4.99% of 500 people 1 point; responses provided by <2% of 500 people 2 points; responses not listed in the technical manual were always given 2 points. The sum of the points across responses was used as the individual originality score.

### The Motor-Form Divergent Thinking Task

**Figure 2:**
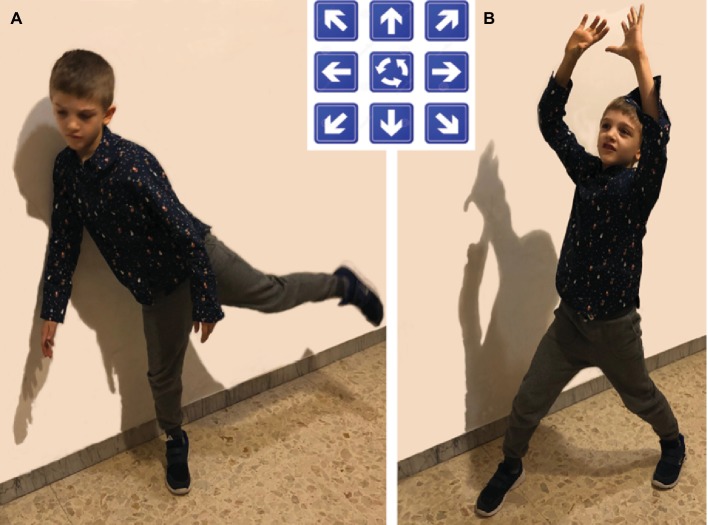
Examples of motor-forms: **(A)** Football player; **(B)** Basketball player. Written informed consent was obtained from the parents of the child represented in the figure specifically for the publication of this image. A copy of the written consent is available for review by the Editor-in-Chief of this journal.

This task was opportunely developed for the present study following the logic underlying the visual divergent thinking task. Participants were asked, without any cues, to perform a free motor action moving from a starting point (from the center of the room) and performing 10 steps in a specific direction (i.e., forward, backward, left, right, diagonally forward left, diagonally forward right, diagonally backward left, diagonally forward right, turn clockwise, and turn counter-clockwise) before executing the invented movement (see Figure 2). Participants had to execute more motor actions as possible within 10 min. At the end of the performed action, children were asked to verbally describe the activity performed with a verb or a substantive. The task performance was not videotaped. One experimenter (observer) took notes of the motor forms performed and the description of the action provided by the child. Motor forms that were not recognizable were discarded at first glance. Afterward, the observer and another experimenter evaluated three basic attributes of motor-form divergent thinking, namely fluency, flexibility, and originality, in order to reach an agreement about them. Since this task was conceived on the basis of the visual divergent thinking task, the scoring procedure was the same. For fluency, the experimenters evaluated the number of the movements produced within the 10-min time limit (thus, the fluency score could be higher than 10). For flexibility, the experimenters generated the categories in which the movement forms fell. Categories were opportunely generated according to the movement forms provided. For example, if the child produced a movement form of a floor cleaner, the category “house work” was generated. In this vein, if a movement form of a football player was produced, the category “athletes” was generated. Thus, if the child produced movement forms still related to sport players, the category “athletes” was used to encompass the forms. For originality, the number of statistically infrequent ideas provided by participants of the present study (58 children) was computed as for the visual divergent thinking task: responses provided by 5% or more of 58 children were scored 0; responses provided by 2–4.99% of 58 children 1 point; responses provided by <2% of 58 children 2 points. The sum of the points across responses was used as the individual originality score.

### Administration of the Tasks

The topological map of the body task, the visual divergent thinking task, and the motor-form divergent thinking task were administered in random order across children. The entire experiment lasted approximately 30 min.

## Results

Means and standard deviations of all variables of interest divided per group are reported in Table 1.

**Table 1:** Descriptive statistics.

	No-dance	Dance
Visual fluency	10 (0)	10 (0)
z-Visual fluency	0 (0)	0 (0)
Visual flexibility	7.56 (1.47)	7.91 (1.40)
z-Visual flexibility	−0.14 (1.03)	0.11 (0.98)
Visual originality	10.36 (2.53)	11.09 (2.24)
z-Visual originality	−0.17 (1.06)	0.13 (0.94)
**Visual creative index**	**−0.31 (1.10)**	**0.24 (1.45)**
Motor fluency	15.56 (3.84)	17.42 (2.15)
z-Motor fluency	−0.34 (1.23)	0.26 (0.69)
Motor flexibility	5.48 (2.14)	7.58 (2.25)
z-Motor flexibility	−0.49 (0.88)	0.37 (0.93)
Motor originality	8.16 (5.27)	14.09 (5.38)
z-Motor originality	−0.56 (0.87)	0.42 (0.89)
**Motor creative index**	**−1.39 (2.39)**	**1.05 (1.84)**

*Means and standard deviations (s.d.) of raw and z-scores in visual and motor creativity taking into consideration fluency, flexibility, originality, and the complete creative index for both types of creativity. The indices are expressed only in z-scores.*

Following the procedure used by Runco et al. (2010), we computed the z-scores of fluency, flexibility, and originality; then, they were summed for obtaining a composite creative index both for visual and motor-form of creative thinking.

In order to exclude effects on visual and motor creativity due to gender, we firstly performed two separate one-way analyses of variance (ANOVA) with gender (girls vs. boys) as independent variable and composite creative indices (visual and motor) as dependent variables. No differences between groups were detected in visual composite creative index (*F*_1,56_ = 1.74; *p* = 0.19; partial eta-square = 0.03; observed power = 0.25) and in motor-form composite creative index (*F*_1,56_ = 1.79; *p* = 0.19; partial eta-square = 0.03; observed power = 0.26).

Thus, since gender produced no effect on the variables of interest, subsequent analyses were carried out focusing only on differences between dancers and non-dancers.

**Figure 3:**
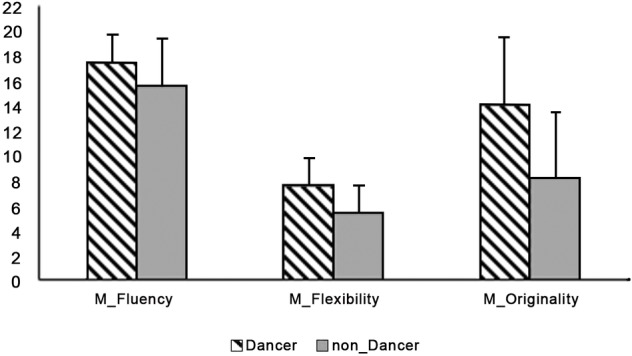
Means and standard deviations of dancers and no dancers in the three motor components of the motor-form divergent thinking task.

We performed two separate one-way analyses of variance (ANOVA) with group (dancers vs. non-dancers) as independent variable and composite creative indices (visual and motor) as dependent variables. No differences between groups were detected in visual composite creative index (*F*_1,56_ = 2.51; *p* = 0.12; partial eta-square = 0.043; observed power = 0.34); on the contrary, the two groups differed in motor-form composite creative index (*F*_1,56_ = 19.29; *p* = 0.000; partial eta-square = 0.26; observed power = 0.99): the dance group outperformed the control group (see Figure 3).

Thus, we performed a separate ANOVA for each score of the motor-form composite creative index (fluency, flexibility, and originality), with group (dancers vs. non-dancers) as the between factor. Results showed that dancers had a significantly better performance in fluency (*F*_1,56_ = 5.51; *p* = 0.022; partial eta-square = 0.09; observed power = 0.64); flexibility (*F*_1,56_ = 12.85; *p* = 0.001; partial eta-square = 0.19; observed power = 0.94); and originality (*F*_1,56_ = 17.58; *p* = 0.000; partial eta-square = 0.24; observed power = 0.99).

We also analyzed time and accuracy in the topological map of the body task as dependent variables comparing the performance of the two groups (dancers vs. non-dancers) as independent variable. Two separate one-way ANOVAs showed that dancers were more accurate (*F*_1,56_ = 8.51; *p* = 0.005; partial eta-square = 0.13; observed power = 0.82) and faster (*F*_1,56_ = 13.86; *p* = 0.000; partial eta-square = 0.20; observed power = 0.96) than non-dancers in localizing the body parts.

**Figure 4:**
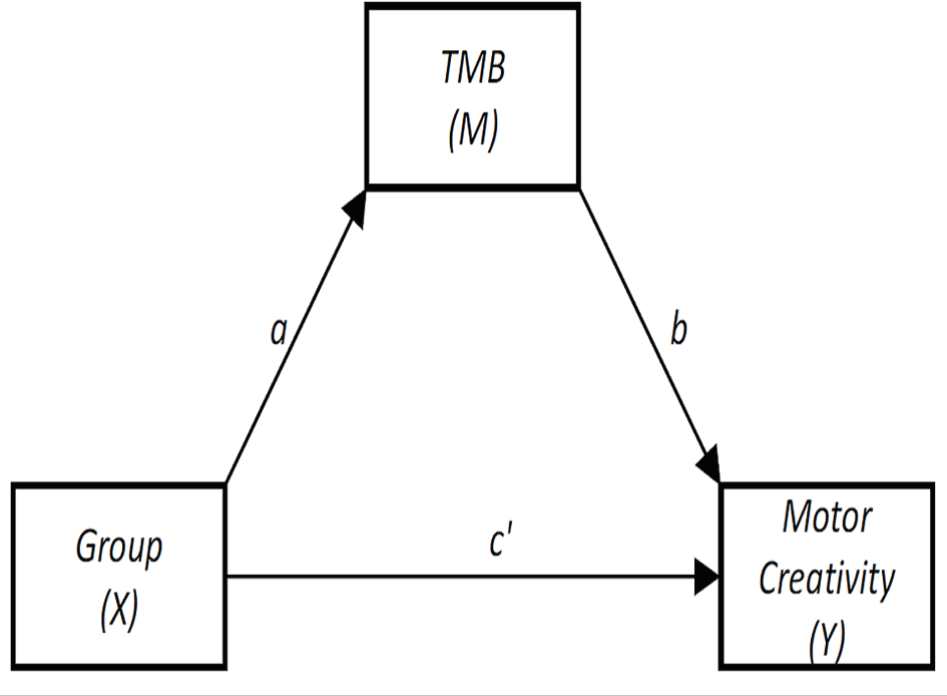
Template of the mediation analysis. Notes: TMB = topological map of the body.

Thus, we directly tested whether the group effect we detected on motor-form of creative thinking was mediated by a more accurate topological map of the body in dancers. Thus, we performed a mediation analysis by using PROCESS (Hayes, 2017) with group (dancers vs. non-dancers) as independent variable (X), motor-form composite creative index as independent variable (Y), and the number of correct answers on TMB task as mediator (M). Figure 4 summarizes this model. We found that Y was significantly predicted both by X (path c′; Figure 4) and M (path b; Figure 4). Also, both direct (path c′) and indirect effects of X on Y were significant. Statistics are fully reported in Table 2.

**Table 2:** Mediation analysis.

Variable	M (TMB)				Y (Motor creativity)			
	Path	*beta*	*t*	*p*	Path	*beta*	*t*	*p*
X (Group)	a	1.3394	2.9175	0.0051	c′	1.8767	3.3249	0.0016
M (TMB)		—	—	—	b	0.4224	2.7595	0.0078
	*R^2^* = 0.1319, *F*_1,56_ = 8.5121, *p* = 0.0051				*R^2^* = 0.3467, *F*_2,55_ = 14.5935, *p* < 0.0001			

*Mediation analysis: effects for mediator (M) and independent variable (X) on motor creativity (Y). The indirect effect of X on Y is significant (0.5658; LLCI = 0.0820, ULCI = 1.1989). The direct effect of X on Y (c) is also significant (R^2^ = 0.2562, F_1,56_ = 19.2929, p = 0.0001; beta = 2.4424 t = 4.3924, p = 0.0001). Notes: TMB = topological map of the body.*

## Discussion

Dance has always been considered a means for creativity and self-expression, allowing the expression of kinetic consciousness (Kalliopuska, 1989; Pavlik and Nordin-Bates, 2016) and enhancing positive self-esteem (Alter, 1984). According to Castañer et al. (2016), kinematic parameters expressed by modern dancers also positively influence the observers’ aesthetic appraisal. In the present study, we were interested in investigating two different aspects. The first one was related to the extent to which dance can promote not only motor but also visual divergent thinking (or creative index). That is, the issue of domain-specificity of divergent thinking was faced using a dance group of children. Contradictory results provided so far by previous studies led us to formulate two hypotheses: first, dance promotes both visual and motor divergent thinking; second, dance promotes only motor divergent thinking. The second aspect investigated was related to the mediation role of the topological map of the body on motor-form divergent thinking. In line with the evidence that the topological map of the body is progressively elaborated on the basis of sensory afferents that, from the beginning of life, maintain a link with motricity (Case-Smith et al., 2001), we hypothesized that the topological map of the body is generally better in non-dancer children. Since the topological map of the body supplies visuospatial information about the online representations of the body parts (Di Vita et al., 2016; Palermo et al., 2018), we hypothesized that the topological map of the body is crucial in enhancing the motor-form divergent thinking.

Results showed that children engaged in a ballet dance program did not differ from the control group in visual composite creative index, but they did in motor-form composite creative index. This finding is consistent with the domain-specific hypothesis of creativity and the evidence that expertise influences cognition selectively. For example, highly skilled poker players have a larger working memory span (Meinz et al., 2012); military pilots are faster at mentally rotating objects (Verde et al., 2013) as well as better in navigational skills (Verde et al., 2016), or may be different in moral dilemmas and decision-making (Boccia et al., 2017); video game players outperform novices on tests of visuospatial attention (Green and Bavelier, 2003). More specifically, for motor domain, exergames—namely, videogames based on body interaction because they foster physical exercise while playing (e.g., the Wii)—were found to elicit motor activities, such as motor endurance (e.g., climbing the stairs), stretch (e.g., in jumps), coordination (e.g., controlling the body throughout the activity), and balance (e.g., keeping the body in stability and in control while moving) (Castañer et al., 2016); yet, high-level athletes were found to report higher levels of vividness of motor imagery (Zhang et al., 2018). With respect to creativity, although expertise and creativity are different constructs, in general, creativity often requires some level of expertise, especially in some domains (Baer, 2015). Consistent with this notion, it is possible that being expert in a specific domain predicts creativity only in that domain but not in others, unrelated domains; in addition, it is also possible that people are experts and creative in many different domains, as well as that one cannot simply transfer expertise and creativity across domains (for a review see Baer, 2015). However, regardless of the level of expertise, given that in adults dance was found to increase fluency (Gondola, 1987) and flexibility (Steinberg et al., 1997) of divergent thinking, it is also possible that in the present study, dance improved only motor divergent thinking because children (6–10 years old) were less flexible in transferring their little expertise to visual domain. Further investigations are needed to explore this issue.

In addition, consistent with our hypotheses, we found that dancers were more accurate and faster in localizing the body parts as compared with non-dancers. This result confirms that the development of the topological map of the body takes advantage from dance practiced already in early childhood. More interestingly, in line with the fourth hypothesis, we found that the topological map of the body mediated the effect of the group on motor-forms divergent thinking: besides the direct effect of group on motor-forms divergent thinking, we detected a significant indirect group effect, which was mediated by performances on TMB task. Therefore, dancers were able to easily generate new movements, to provide unique and rare movements as well as to change categories of movements with respect to non-dancers, at least in part due to their improved topological map of the body.

In conclusion, in line with the worldwide health organizations, that suggested to increase levels of physical activity in school-aged children to enhance emotional, social, and cognitive functioning across their life span, the present findings support the idea to perform dance during early development because it affects many aspects of children’s health (King et al., 2003) and may also have important implications for cognition, such as motor divergent thinking and creativity. The evidence that performing dance may enhance motor creativity, specifically the ability to produce motor forms, is interesting because it highlights the key role of this kind of physical activity in children’s life and the need to perform it throughout adulthood (Tucker, 2008). In addition, when improvisation is pursued, such as in modern dance (Fink and Woschnjak, 2011) or free movements with music (Campion and Letiva, 2014), it can be expected that dance produces facilitation effects also in other domains. Further studies are necessary to clarify this issue.

Regarding limitations, the absence of validation of the motor-form divergent thinking task limits the conclusions drawn by the present study. The scarce number of participants and the gender imbalance in the dance group should also be considered as possible confounding factors, even though it is noteworthy to underline that it is not easy to find children that practice classical ballet, especially boys. In future, studies with more participants based on different dance styles, equally practiced by both genders, should be pursued. In the future, given the nature of the motor-form divergent thinking task, videorecording is suggested in order to ensure that observers have observed movements properly. This would also help to evaluate movements by multiple independent judges and get the intra- and inter-agreement within and between raters, respectively. Despite these limitations, this study involves two novelties: the exploration of the relation between dance and divergent thinking in different forms (motor and visual) in children; the exploration of the extent to which the topological map of the body mediates motor divergent thinking in dancer children. Moreover, the present results also have important implications for creative dance in educational settings, because dance has to be conceived as a healing and aesthetic art (Thomson, 2011) since childhood. Following Castañer et al. (2009), it could be interesting to compare the impact of different kinds of instructions on motor creativity. Authors refer to descriptive instructions: questions using specific terminology of dance; metaphoric instructions: questions using metaphorical images; and kinesic instructions: motor examples or visual demonstrations. Descriptive and, to a lesser extent, metaphoric instructions were found to generate more motor divergence than kinesic instructions. Metaphoric instructions were found to help to understand the task and motivate participants. Therefore, Castañera and colleagues suggested to use kinesic and metaphoric instructions with beginners, and descriptive instructions with more expert dancers. According to Davenport (2006), among other teaching points, it might be also useful to translate ideas into movements of the body and develop different body movements of the same idea, draw on improvisation, learn to feel the body (e.g., energy, body parts initiation), manipulate chunks of movements, vary rhythm and length of movements, translate the notion of outside eye to inner eye to better understand what movements are expressing and develop different kinds of relationships with the co-dancers. In other words, the pedagogical value of creative dance has to be pursued using different dance styles because it has the potential to offer the opportunity to develop well-being, general cognition, and wider creativity, besides contributing to the improvement of bodily self-consciousness and motor creativity.

## Ethics Statement

The study was approved by the Ethics Committee of University of L’Aquila in accordance with the Declaration of Helsinki. A signed informed consent was obtained from parents and an assent from each child.

## Author Contributions

MP has projected the study and has written the paper. LG has helped in projecting the study and has collected data. PG has managed data and has contributed to writing the paper. MB has helped in data analysis. SD has helped in writing the paper. LP has managed data and has written the paper.

### Conflict of Interest Statement

The authors declare that the research was conducted in the absence of any commercial or financial relationships that could be construed as a potential conflict of interest.

## References

[ref1] Alter J. B. (1984). Creativity profile of university and conservatory dance students. J. Pers. Assess. 48, 153–158. 10.1207/s15327752jpa4802_8 16367539

[ref2] Alter J. (1999). “Dance and creativity” in Encyclopedia of creativity. eds. Runco M. A. Pritzker S. R. (London: Academic Press), 496–481.

[ref3] Ayob A. Hussain A. Mustaffa M. M. Kajid R. A. (2012). Assessment of creativity in electrical engineering. Procedia Soc. Behav. Sci. 60, 463–467. 10.1016/j.sbspro.2012.09.407

[ref5] Baer J. (2015). The importance of domain-specific expertise in creativity. Roeper Rev. 37, 165–178. 10.1080/02783193.2015.1047480

[ref6] Barnikel F. Ellbrunner H. Vetter M. (2014). Teaching spatial competence today—from analogue maps to geocaching. Cartogr. J. 5, 257–262.

[ref7] Bassolino M. Finisguerra A. Canzoneri E. Serino A. Pozzo T. (2015). Dissociating effect of upper limb non-use and overuse on space and body representations. Neuropsychologia 70, 385–392. 10.1016/j.neuropsychologia.2014.11.028, PMID: 25462198

[ref8] Boccia M. Piccardi L. Palermo L. Nori R. Palmiero M. (2015). Where do bright ideas occur in our brain? Meta-analytic evidence from neuroimaging studies of domain-specific creativity. Front. Psychol. 6:1195. 10.3389/fpsyg.2015.01195, PMID: 26322002 PMC4531218

[ref9] Boccia M. Verde P. Angelino G. Carrozzo P. Vecchi D. Piccardi L.. (2017). Effect of professional expertise and exposure to everyday life decision-making on moral choices. Neurosci. Lett. 654, 80–85. 10.1016/j.neulet.2017.06.036, PMID: 28647290

[ref10] Brennan M. A. (1982). Relationship between creative ability in dance and selected creative attributes. Percept. Mot. Skills 55, 47–56. 10.2466/pms.1982.55.1.47

[ref11] Campion M. Letiva L. (2014). Enhancing positive affect and divergent thinking abilities: play some music and dance. J. Posit. Psychol. 9, 137–145. 10.1080/17439760.2013.848376

[ref12] Cannoni E. Tega A. (2009). Changes of body schema in milddle childhood. [Cambiamenti dello schema corporeo nella media fanciullezza]. Rass. Psicol 3, 131–140. 10.7379/70585

[ref13] Canzano L. Piccardi L. Bureca I. Guariglia C. (2011). Mirror writing resulting from an egocentric representation disorder: a case report. Neurocase 17, 447–460. 10.1080/13554794.2010.532143, PMID: 21830864

[ref14] Cardinali L. Brozzoli C. Farné A. (2009). Peripersonal space and body schema: two labels for same concept. Brain Topogr. 21, 252–260. 10.1007/s10548-009-0092-7, PMID: 19387818

[ref15] Case-Smith J. Allen A. S. Pratt P. N. (2001). Occupational therapy for children. 4th Edn. (St. Louis: Mosby).

[ref18] Castañer M. Camerino O. Landry P. Parés N. (2016). Quality of physical activity of children in exergames: sequential body movement analysis and its implications for interaction design. Int. J. Hum. Comput. Stud. 96, 67–78. 10.1016/j.ijhcs.2016.07.007

[ref16] Castañer M. Torrents C. Anguera M. T. Dinušová M. Jonsson G. K. (2009). Identifying and analyzing motor skill responses in body movement and dance. Behav. Res. Methods 41, 857–867. 10.3758/BRM.41.3.857, PMID: 19587202

[ref17] Castañer M. Torrents C. Morey G. Jofre T. (2012). “Appraising choreographic creativity, aesthetics and the complexity of motor responses in dance” in Mixed methods research in the movement sciences: Case studies in sport, physical education and dance. eds. Camerino O. Castañer M. Anguera M. T. (Abingdon: Routledge), 146–176.

[ref19] Cimmino R. L. Spitoni G. Serino A. Antonucci G. Catagni M. Camagni M.. (2013). Plasticity of body representations after surgical arm elongation in an achondroplasic patient. Restor. Neurol. Neurosci. 31, 287–298. 10.3233/RNN-120286, PMID: 23396370

[ref20] Couillandre A. Lewton-Brain P. Portero P. (2008). Exploring the effects of kinesiological awareness and mental imagery on movement intention in the performance of demi-plié. J. Dance Med. Sci. 12, 91–98. PMID: 19618584

[ref21] Damasio A. (2010). Self comes to mind: Constructing the conscious brain. (New York: Pantheon Books).

[ref22] Daurat-Hmeljiak C. Stambak M. Berges J. (1978). Il test dello schema corporeo. Una prova di conoscenza e costruzione dell’immagine del corpo [The body schema test. A test of knowledge and construction of body image]. (Firenze, Italy: Organizzazioni Speciali).

[ref23] Davenport D. (2006). Building a dance composition course: an act of creativity. J. Dance Edu. 6, 25–32. 10.1080/15290824.2006.10387309

[ref24] Di Russo F. Committeri G. Pitzalis S. Spitoni G. Piccardi L. Galati G.. (2006). Cortical plasticity following surgical extension of lower limbs. NeuroImage 30, 172–183. 10.1016/j.neuroimage.2005.09.051, PMID: 16288893

[ref26] Di Vita A. Boccia M. Palermo L. Guariglia C. (2016). To move or not to move, that is the question! Body schema and non-action oriented body representations: an fMRI meta- analytic study. Neurosci. Biobehav. Rev. 68, 37–46. 10.1016/j.neubiorev.2016.05.005, PMID: 27177829

[ref28] Di Vita A. Palermo L. Boccia M. Guariglia C. (2019). Topological map of the body in post-stroke patients: lesional and hodological aspects. Neuropsychology. 10.1037/neu0000536, PMID: (in press). 30730163

[ref27] Di Vita A. Palermo L. Piccardi L. Di Tella J. Propato F. Guariglia C. (2017). Body representation alterations in personal but not in extrapersonal neglect patients. Appl. Neuropsychol. Adult 24, 308–317. 10.1080/23279095.2016.1174866 27183152

[ref25] Di Vita A. Palermo L. Piccardi L. Guariglia C. (2015). Peculiar body representation alterations in hemineglect: a case report. Neurocase 21, 697–706. 10.1080/13554794.2014.974620 25360817

[ref29] Ellbrunner H. Barnikel F. Vetter M. (2014). “Geocaching as a method to improve not only spatial but also social skills—Results from a school project” in GI_Forum 2014-Geospatial Innovation for Society. eds. Vogler R. Car A. Strobl J. Griesebner G. (Berlin: Wichmann), 348–351.

[ref31] Fink A. Graif B. Neubauer A. C. (2009). Brain correlates underlying creative thinking: EEG alpha activity in professional vs. novice dancers. NeuroImage 46, 854–862. 10.1016/j.neuroimage.2009.02.036, PMID: 19269335

[ref30] Fink A. Woschnjak S. (2011). Creativity and personality in professional dancers. Pers. Individ. Differ. 51, 754–758. 10.1016/j.paid.2011.06.024

[ref33] Fonseca C. C. Thurm B. E. Vecchi R. L. (2014). Ballroom dance and body size perception. Percept. Mot. Skills 119, 495–503. 10.2466/25.PMS.119c26z1, PMID: 25349891

[ref32] Fonseca C. C. Vecchi R. L. Gama E. F. (2012). The ballroom dance influence in body perception. Motriz: Rev. Educ. Fis. 18, 200–207. 10.1590/S1980-65742012000100020

[ref34] Fuentes C. T. Pazzaglia M. Longo M. R. Scivoletto G. Haggard P. (2013). Body image distortions following spinal cord injury. J. Neurol. Neurosurg. Psychiatry 84, 201–207. 10.1136/jnnp-2012-304001, PMID: 23204474

[ref35] Gondola J. C. (1987). The effects of a single bout of aerobic dancing on selected tests of creativity. J. Soc. Behav. Pers. 2, 275–278.

[ref36] Green C. S. Bavelier D. (2003). Action video game modifies visual selective attention. Nature 423, 534–537. 10.1038/nature01647, PMID: 12774121

[ref37] Guariglia C. Piccardi L. Puglisi Allegra M. C. Traballesi M. (2002). Is autotopoagnosia real? EC says yes. A case study. Neuropsychologia 40, 1744–1749. 10.1016/S0028-3932(02)00013-1, PMID: 11992662

[ref38] Guilford J. P. (1950). Creativity. Am. Psychol. 5, 444–454. 10.1037/h0063487, PMID: 14771441

[ref39] Guilford J. P. (1967). The nature of human intelligence. (New York, NY: McGraw-Hill).

[ref40] Guilford J. P. Christensen P. R. Merrifield P. R. Wilson R. C. (1978). Alternate uses: Manual of instructions and interpretation. (Orange, CA: Sheridan Psychological Services).

[ref41] Hayes A. F. (2017). Introduction to mediation, moderation, and conditional process analysis second edition. A regression-based approach. (New York, NY: Guilford Press).

[ref42] Hillman C. H. Biggan J. R. (2017). A review of childhood physical activity, brain, and cognition: perspectives on the future. Pediatr. Exerc. Sci. 29, 170–176. 10.1123/pes.2016-0125, PMID: 27615274

[ref43] Humble S. Dixon P. Mpofu E. (2018). Factor structure of the Torrance Test of Creative Thinking Figural Form A in Kiswahili speaking children: multidimensionality and influences on creative behaviour. Think. Skills Creat. 27, 33–44. 10.1016/j.tsc.2017.11.005

[ref44] Iivonen K. S. Sääkslahti A. K. Mehtälä A. Villberg J. J. Tammelin T. H. Kulmala J. S.. (2013). Relationship between fundamental motor skills and physical activity in 4-year-old preschool children. Percept. Mot. Skills 117, 627–646. 10.2466/10.06.PMS.117x22z7, PMID: 24611263

[ref45] Kalliopuska M. (1989). Empathy, self-esteem and creativity among junior ballet dancers. Percept. Mot. Skills 69, 1277–1234. 10.2466/pms.1989.69.3f.1227 2622738

[ref46] Kaufman J. C. Baer J. (2005). Creativity across domains: Faces of the muse. (Mahwah, NJ: Lawrence Erlbaum Associates).

[ref47] King G. Law M. King S. Rosenbaum P. Kertoy M. K. Young N. L. (2003). A conceptual model of the factors affecting the recreation and leisure participation of children with disabilities. Phys. Occup. Ther. Geriatr. 23, 63–90. 10.1080/J006v23n01_05 12703385

[ref48] Lewis R. N. Scannell E. D. (1995). Relationship of body image and creative dance movement. Percept. Mot. Skills 81, 155–160. 10.2466/pms.1995.81.1.155 8532452

[ref49] Lis A. Tallandini M. (1981). “Taratura italiana delle prove” in Il test dello Schema Corporeo. Una prova di conoscenza e di costruzione dell’immagine del corpo. eds. Daurat-Hmeljak C. Stambak M. Bergès J. (Firenze: Organizzazioni Speciali).

[ref51] Marangolo P. Piccardi L. Rinaldi M. C. (2003). Dissociation between personal and extrapersonal neglect in a crossed aphasia study. Neurocase 9, 414–420. 10.1076/neur.9.5.414.16554 14972756

[ref52] Meinz E. J. Hambrick D. Z. Hawkins C. B. Gillings A. K. Meyer B. E. Schneider J. L. (2012). Roles of domain knowledge and working memory capacity in components of skill in Texas Hold'Em poker. J. Appl. Res. Mem. Cogn. 1, 34–40. 10.1016/j.jarmac.2011.11.001

[ref53] Moraru A. Memmert D. van der Kamp J. (2016). Motor creativity: the roles of attention breadth and working memory in a divergent doing task. J. Cogn. Psychol. 28, 856–867. 10.1080/20445911.2016.1201084

[ref54] Ozel S. Larue J. Molinaro C. (2002). Relation between sport activity and mental rotation: comparison of three groups of subjects. Percept. Mot. Skills 95, 1141–1154. 10.2466/pms.2002.95.3f.1141 12578254

[ref55] Paillard J. (1980). Le corps situé et le corps identifie: une approche psychophysyiologique de la notion de schema corporel. Rev. Med. Suisse 100, 129–141. 6769144

[ref57] Palermo L. Di Vita A. Boccia M. Nemmi F. Brunelli S. Traballesi M.. (2018). Action and non-action oriented body representations: insight from behavioural and grey matter modifications in individuals with lower limb amputation. Biomed. Res. Int. 2018, 1529730. 10.1155/2018/1529730, PMID: 30420956 PMC6211209

[ref56] Palermo L. Di Vita A. Piccardi L. Traballesi M. Guariglia C. (2014). Bottom-up and top-down processes in body representation: a study of brain-damaged and amputee patients. Neuropsychology 28, 772–781. 10.1037/neu0000086, PMID: 24799290

[ref60] Palmiero M. Di Giacomo D. Passafiume D. (2016a). Can creativity predict cognitive reserve? J. Creat. Behav. 50, 7–23. 10.1002/jocb.62

[ref58] Palmiero M. Nakatani C. Raver D. Olivetti Belardinelli M. vanLeeuwen C. (2010). Abilities within and across visual and verbal domains: how specific is their influence on creativity? Creat. Res. J. 22, 369–377. 10.1080/10400419.2010.523396

[ref59] Palmiero M. Nori R. Aloisi V. Ferrara M. Piccardi L. (2015). Domain-specificity of creativity: a study on the relationship between visual creativity and visual mental imagery. Front. Psychol. 6:1870. 10.3389/fpsyg.2015.01870, PMID: 26648904 PMC4664616

[ref61] Palmiero M. Nori R. Piccardi L. (2016b). Visualizer cognitive style enhances visual creativity. Neurosci. Lett. 615, 98–101. 10.1016/j.neulet.2016.01.032 26806864

[ref62] Palmiero M. Nori R. Piccardi L. (2017). Verbal and visual divergent thinking in aging. Exp. Brain Res. 235, 1021–1029. 10.1007/s00221-016-4857-4, PMID: 28032140

[ref63] Pavlidou E. Sofianidou A. Lokosi A. Kosmidou E. (2018). Creative dance as a tool for developing preschoolers’ communicative skills and movement expression. Eur. Psychomot. J. 10, 3–15.

[ref64] Pavlik K. Nordin-Bates S. (2016). Imagery in dance. A literature review. J. Dance Med. Sci. 20, 51–63. 10.12678/1089-313X.20.2.51, PMID: 27245944

[ref65] Perez-Marcos D. Martini M. Fuentes C. T. Bellido Rivas A. L. Haggard P. Sanchez-Vives M. V. (2018). Selective distortion of body image and asynchronous visuotactile stimulation. Body Image 24, 55–61. 10.1016/j.bodyim.2017.11.002, PMID: 29268137

[ref66] Piaget J. (1952). The origins of intelligence in children. (Cook M., Trans.). (New York, NY, US: W W Norton & Co).

[ref67] Plucker J. A. Beghetto R. A. (2004). “Why creativity is domain general, why it looks domain specific, and why the distinction does not matter” in Creativity: From potential to realization. eds. Sternberg R. J. Grigorenk E. L. Singer J. L. (Washington, DC: American Psychological Association), 153–167.

[ref68] Purcell T. M. (1990). The use of imagery in children’s dance: making it work. J. Phys. Educ. Rec Dance 61, 22–23. 10.1080/07303084.1990.10606436

[ref69] Runco M. A. Millar G. Acar S. Cramond B. (2010). Torrance tests of creative thinking as predictors of personal and public achievement: a fifty-year follow-up. Creat. Res. J. 22, 361–368. 10.1080/10400419.2010.523393

[ref70] Sacha T. J. Russ S. W. (2006). Effects of pretend imagery on learning dance in preschool children. Early Childhood Educ. J. 33, 341–345. 10.1007/s10643-006-0103-1

[ref71] Schmidt M. Egger F. Kieliger M. Rubeli B. Schuler J. (2016). Gymnasts and orienteers display better mental rotation performance than non athletes. J. Individ. Differ. 37, 1–7. 10.1027/1614-0001/a000180

[ref72] Schwoebel J. Coslett H. B. (2005). Evidence for multiple, distinct representations of the human body. J. Cogn. Neurosci. 17, 543–553. 10.1162/0898929053467587, PMID: 15829076

[ref73] Scibinetti P. Tocci N. Pesce C. (2011). Motor creativity and creative thinking in children: the diverging role of inhibition. Creat. Res. J. 23, 262–272. 10.1080/10400419.2011.595993

[ref75] Silvia P. J. (2008a). Another look at creativity and intelligence: exploring higher-order models and probable confounds. Pers. Individ. Differ. 44, 1012–1021. 10.1016/j.paid.2007.10.027

[ref76] Silvia P. J. (2008b). Creativity and intelligence revisited: a latent variable analysis of Wallach and Kogan (1965). Creat. Res. J. 20, 34–39. 10.1080/10400410701841807

[ref74] Silva P. J. Kaufman J. C. Pretz J. E. (2009). Is creativity domain-specific? Latent class models of creative accomplishments and creative self-descriptions. Psychol. Aesthet. Creat. Arts 3, 139–148. 10.1037/a0014940

[ref77] Singh A. Uijtdewilligen L. Twisk J. W. van Mechelen W. Chinapaw M. J. (2012). Physical activity and performance at school. A systematic review of the literature including a methodological quality assessment. Arch. Pediatr. Adolesc. Med. 166, 49–55. 10.1001/archpediatrics.2011.716, PMID: 22213750

[ref78] Spitoni G. F. Serino A. Cotugno A. Mancini F. Antonucci G. Pizzamiglio L. (2015). The two dimensions of the body representation in women suffering from Anorexia Nervosa. Psychiatry Res. 230, 181–188. 10.1016/j.psychres.2015.08.036, PMID: 26360978

[ref79] Sprini G. Tomasello S. (1989). Torrance tests of creative thinking (Test di pensiero Creativo). (Firenze: Giunti O.S. Organizzazioni Speciali).

[ref80] Steinberg H. Sykes E. A. Moss T. Lowerey S. LeBoutillier N. Dewey A. (1997). Exercise enhances creativity indipendently of mood. Br. J. Sports Med. 31, 240–245. 10.1136/bjsm.31.3.240, PMID: 9298561 PMC1332529

[ref81] Stinson S. W. (1993). Testing creativity of dance students in the people republic of china. Dance Res. J. 25, 65–68. 10.1017/S0149767700008056

[ref82] Thomson P. (2011). “Dance and creativity” in Encyclopedia of creativity. eds. Runco M. A. Pritzker S. R. (London: Academic Press), 334–350.

[ref83] Thurm B. E. Pereira E. S. Fonseca C. C. Cagno M. J. S. Gama E. F. (2011). Neuroanatomical aspects of the body awareness. J. Morphol. Sci. 28, 296–299.

[ref84] Torrance E. P. (1987). Guidelines for administration and scoring comments on using the Torrance Tests of Creative Thinking. (Bensenville, IL: Scholastic Testing Service Inc).

[ref85] Tucker P. (2008). The physical activity levels of preschool-aged children: a systematic review. Early Child Res. Q. 23, 547–558. 10.1016/j.ecresq.2008.08.005

[ref87] Verde P. Boccia M. Colangeli S. Barbetti S. Nori R. Ferlazzo F.. (2016). Domain-specific interference tests on navigational working memory in military pilots. Aerosp. Med. Hum. Perf. 87, 528–533. 10.3357/AMHP.4521.2016, PMID: 27208675

[ref86] Verde P. Piccardi L. Bianchini F. Trivelloni P. Guariglia C. Tomao E. (2013). Gender effects on mental rotation in pilots vs. nonpilots. Aviat. Space Environ. Med. 84, 726–729. 10.3357/ASEM.3466.2013, PMID: 23855069

[ref88] Vignemont F. (2010). Body schema and body image: pros and cons. Neuropsychologia 48, 669–680. 10.1016/j.neuropsychologia.2009.09.022, PMID: 19786038

[ref89] Whitehead M. E. (2010). Physical literacy: Throughout the lifecourse. (London: Routledge).

[ref90] Wyrick W. (1968). The development of a test of motor creativity. Res. Q. 39, 756–765. 10.1080/10671188.1968.10616608, PMID: 5246984

[ref91] Zantedeschi M. Pazzaglia M. (2016). Commentary: non-invasive brain stimulation, a tool to revert maladaptive plasticity in neuropathic pain. Front. Hum. Neurosci. 10:544. 10.3389/fnhum.2016.00544, PMID: 27833544 PMC5081357

[ref92] Zeng N. Ayyub M. Sun H. Wen X. Xiang P. Gao Z. (2017). Effects of physical activity on motor skills and cognitive development in early childhood: a systematic review. Biomed. Res. Int. 2760716, 1–13. 10.1155/2017/2760716 PMC5745693 29387718

[ref93] Zhang L. Pi Y. Zhu H. Shen C. Zhang J. Wu Y. (2018). Motor experience with a sport-specific implement affects motor imagery. PeerJ 6:e4687. 10.7717/peerj.4687, PMID: 29719738 PMC5926550

