# Corrigendum: The Dancers' Visuospatial Body Map Explains Their Enhanced Divergence in the Production of Motor Forms: Evidence in the Early Development

**DOI:** 10.3389/fpsyg.2019.01274

**Published:** 2019-05-28

**Authors:** Massimiliano Palmiero, Luna Giulianella, Paola Guariglia, Maddalena Boccia, Simonetta D'Amico, Laura Piccardi

**Affiliations:** ^1^Cognitive and Motor Rehabilitation and Neuroimaging Unit, IRCCS Fondazione Santa Lucia, Rome, Italy; ^2^Department of Biotechnological and Applied Clinical Sciences, University of L'Aquila, L'Aquila, Italy; ^3^Life, Health and Environmental Science Department, University of L'Aquila, L'Aquila, Italy; ^4^Department of Human Science and Society, Kore University of Enna, Enna, Italy

**Keywords:** dance, divergent thinking, creativity, visual, motor, verbal, education, expertise

In the published article, there was an error regarding the affiliation for Maddalena Boccia. Instead of affiliation “5,” it should be “1.”

The authors apologize for this error and state that this does not change the scientific conclusions of the article in any way. The original article has been updated.

